# A Pharmacological and Toxicological Profile of Silver as an Antimicrobial Agent in Medical Devices

**DOI:** 10.1155/2010/910686

**Published:** 2010-08-24

**Authors:** Alan B. G. Lansdown

**Affiliations:** Division of Investigative Medicine, Faculty of Medicine, Imperial College, London W6 8RP, UK

## Abstract

Silver is used widely in wound dressings and medical devices as a broad-spectrum antibiotic. Metallic silver and most inorganic silver compounds ionise in moisture, body fluids, and secretions to release biologically active Ag^+^. The ion is absorbed into the systemic circulation from the diet and drinking water, by inhalation and through intraparenteral administration. Percutaneous absorption of Ag^+^ through intact or damaged skin is low. Ag^+^ binds strongly to metallothionein, albumins, and macroglobulins and is metabolised to all tissues other than the brain and the central nervous system. Silver sulphide or silver selenide precipitates, bound lysosomally in soft tissues, are inert and not associated with an irreversible toxic change. Argyria and argyrosis are the principle effects associated with heavy deposition of insoluble silver precipitates in the dermis and cornea/conjunctiva. Whilst these changes may be profoundly disfiguring and persistent, they are not associated with pathological damage in any tissue. The present paper discusses the mechanisms of absorption and metabolism of silver in the human body, presumed mechanisms of argyria and argyrosis, and the elimination of silver-protein complexes in the bile and urine. Minimum blood silver levels consistent with early signs of argyria or argyrosis are not known. Silver allergy does occur but the extent of the problem is not known. Reference values for silver exposure are discussed.

## 1. Introduction

Silver is a white lustrous transitional metallic element found widely in the human environment. Low concentrations of silver are present in the human body through inhalation of particles in the air and contamination of the diet and drinking water, but silver serves no trace metal value in the human body. Increasing use of silver as an efficacious chemotherapeutic antibacterial and antifungal agent in wound care products, medical devices (bone cements, catheters, surgical sutures, cardiovascular prostheses, and dental fillings), textiles, cosmetics, and even domestic appliances in recent years has lead to concern as to the safety aspects of the metal and potential risks associated with the absorption of the biologically active Ag^+^ into the human body [1]. Safety thresholds set by regulatory authorities including the World Health Organisation and U.S. Environmental Protection Agency are based mostly on scientific reports conducted before the introduction of the high standards of experimental and investigative procedures and tissue analysis expected nowadays and fail to recognise more recent work [2, 3].

Metallic silver is inert in the presence of human tissues but ionises in the presence of moisture, body fluids, and secretions to release the biologically active Ag^+^ which shows a strong affinity for sulphydryl groups and other anionic ligands of proteins, cell membranes, and tissue debris [4]. Ionisation of metallic silver is proportional to the surface area of particles; release of Ag^+^ from nanocrystalline particles of <20 nm being more than one-hundred-fold higher than that from silver foil or other metallic silver forms. Comparative studies have shown that nanocrystalline silver with higher solubility in water exhibits a sixfold or higher log reduction in *Pseudomonas aeruginosa *in culture [4]. Ag^+^ binds protein residues on cell membranes of sensitive bacteria, fungi, and protozoa and is absorbed intracellularly by pinocytosis. Subsequent denaturation and inactivation of proteins and essential enzymes including RNA- and DNA-ases forms the basis of the genetically regulated antimicrobial action of silver [5, 6]. Silver-sensitive strains of bacteria and fungi have been shown to absorb and concentrate Ag^+^ from dilute solutions (1 ppm) by an oligodynamic action, first described by the Swiss botanist Von Nägeli in 1893 [7]. Experimental studies suggest that concentrations of 60 ppm Ag^+^ should be sufficient to control the majority of bacterial and fungal pathogens [4].

Recent advances in the biotechnology of medical devices and the ability to impregnate or coat alginates, polyurethane, silicones, and textile fibres with ionisable silver compounds now provide clinicians with efficacious means of overcoming infections in wound care and device-related infections which have proved costly in terms of hospital care and patient stress as well as being a major cause for fatalities [8–10]. Wound dressings, catheters, bone cements, dental devices, hygiene textiles, consumer products, and other products area treated with silver as antibiotic or preservative release bioactive Ag^+^ to achieve antibacterial or antifungal action. Limited evidence is available currently to show that nanoparticulate silver is an efficacious antiviral agent [11, 12]. Lara et al. have shown that silver nanoparticles exert antiviral action against HIV-1 at noncytotoxic concentrations, but these mechanisms of action which have not been fully elucidated involve virion binding, inhibition of replication, and inactivation [11].

Silver should be classified as a xenobiotic metal in the human body. Available evidence suggests that much of the ion released precipitates with chloride or phosphate anions or becomes strongly bound in the form of inert complexes with albumins or macroglobulins; some binds or is deposited in tissue debris [13, 14]. This bound ion is not available for antibiotic purposes but is of potential significance as a toxic factor [4, 15].

Clinical studies with antibiotic wound dressings have shown that most of the Ag^+^ released into the wound bed is deposited superficially and that minimal levels are available for absorption [16]. Proteins in the systemic circulation and at sites of contact avidly bind the lower concentrations of Ag^+^ released from antibiotic in in-dwelling catheters, cardiovascular devices, and orthopaedic cements, pins, and fixation materials. Occupational health studies demonstrate that greatest risk of silver absorption is anticipated following chronic exposures to silver and silver oxide dusts or silver nitrate particles or aerosol droplets, each of which has been associated with deposition of inert silver sulphide or silver selenide precipitates in the form of argyria and argyrosis in the skin (dermis) and eye (cornea and conjunctiva), respectively [17, 18]. Argyria may present as a profound cosmetic disfigurement which is not readily removed by surgical (dermabrasion) or chemical means [19], but it is not associated with tissue damage or dysfunction. Long-term usage of unregulated and medically unsupervised colloidal silver formulations marketed as nasal decongestants, therapies for allergic rhinitis, and other complaints of infective and noninfectious aetiology are well documented causes of argyria [19–21].

The anti-inflammatory effects of silver nitrate or nanocrystalline silver have been recognised experimentally in wound care, treatment of allergic contact dermatitis ulcerative colitis, and cystitis [22–26]. In dinitrochlorobenzene-induced porcine or murine skin decreased inflammation following application of nanocrystalline silver was associated with lymphocyte apoptosis, decreased expression of pro-inflammatory cytokines, and reduced gelatinase activity. Silver nitrate (0.5%) evoked a wider level of cellular apoptosis but delayed wound healing. In a rat model of ulcerative colitis, administration of 4 mg·kg^−1^ nanocrystalline silver intracolonically or 40 mg·kg^−1^ orally significantly reduced inflammatory changes, partly through suppression of matrix metalloproteinase (MMP-9), tumour necrosis factor (TNF), and interleukin-*β* (IL-*β*) and IL-12 [24]. Other anti-inflammatory changes associated with intravesicular administration of nanocrystalline silver in murine cystitis included suppression of mast cells and urine histamine levels [25].

Wright et al. examining early events in a porcine wound-healing model noted that nanocrystalline silver in particular as used in wound dressings was an efficacious antibacterial agent and significantly promoted wound healing with rapid neovascularisation and suppression of metalloproteinases without compromising other essential events in the wound healing cascade [26]. Further studies are now indicated to confirm this action of nanocrystalline silver in human wound repair.

The toxicology of silver is not well documented and much of the available information concerning the release of Ag^+^ from medical devices and other products intended for human use is ambiguous and widely scattered. Few relevant experimental studies in animal models are reported openly; whilst insufficient in predicting human risk from silver exposure, they do provide relevant information on cytotoxicity, intracellular management of Ag^+^, and routes of excretion [1, 14, 27–29]. It is my intention here to highlight progress on the safety assessment of silver in recent years and to emphasise the gaps in existing knowledge relevant to establishing realistic safety thresholds.

## 2. Absorption and Metabolism of Silver

Expressions of toxicity for any xenobiotic material are related directly to the amount absorbed into the body, its metabolism and accumulation in target organs, and cellular vulnerability to irreversible toxic change. Clinical and experimental studies have shown that metals absorbed into the body interact and compete for binding sites on carrier proteins, and that when protective mechanisms afforded by key metal-binding proteins like metallothioneins and the epidermal barrier function become saturated, toxic changes occur [30–32]. Silver is absorbed into the human body through ingestion, inhalation, intraparenteral insertion of medical devices, and through dermal contact, but the literature on silver absorption by all routes in humans is fragmentary, poorly correlated, and not strong statistically. Metabolic pathways are similar irrespective of route of uptake [1]. Much information on the uptake of silver as a cause of argyria and raised blood silver is derived from occupational health studies where workers have been exposed to silver and silver compounds over many years [3, 17, 33, 34]. From this data it is rarely possible to identify how much silver is absorbed into the circulation from the gastrointestinal tract, lungs, or percutaneous absorption or how much is retained, but urinary or faecal silver excretion may be informative [17, 35–37]. The maximum carrying capacity of human blood for silver is not known but is expected to relate to albumin and macroglobulin concentrations. Armitage et al. studied the uptake of silver in the blood of workers exposed occupationally to silver and reported levels ranging from 0.1 to 23.0 *μ*g·L^−1^ with the highest levels in silver reclaimers [38]. Few objective studies on silver uptake and excretion are reported but Di Vincenzo et al. noted that in 37 workers exposed to silver in smelting and refining, mean silver concentrations in blood, urine, and faeces were 11 *μ*g·L^−1^, <0.005 *μ*g·g^−1^, and 15 *μ*g·g^−1^, respectively [17]. Hair concentrations of silver were markedly higher at 130 ± 160 *μ*g·g^−1^ compared to 0.57 ± 0.56 *μ*g·g^−1^ in controls.

### 2.1. Oral Administration and Gastrointestinal Absorption

Principle routes for buccal or gastrointestinal absorption of silver include

- contaminated food,
- occupational exposures to metallic silver dust, silver oxide, and silver nitrate aerosols,
- drinking water (including use of silver : copper filters in water purification),
- silver nitrate or colloidal silver therapies in oral hygiene and gastrointestinal infection,
- colloidal silver preparations labelled as “food supplements” or “alternative medicines”,
- silver acetate antismoking therapies,
- silver amalgams used in dentistry,
- accidental consumption of silver nitrate or other colourless silver compounds.

Silver absorption through buccal membranes and gastrointestinal mucosae is determined by the ionisation of the silver source and availability of “free” Ag^+^ to interact with protein receptors on cell membranes. Passive uptake is not indicated on account of the high reactivity of the silver ion and its binding capacity of sulphydryl, carboxyl, hydroxyl, and protein ligands on mucosal surfaces and cell debris. Biologically active Ag^+^ readily binds and precipitates with organic constituents of food (phytate, fibres, etc.) and inorganic cations like chloride and phosphate greatly, thereby reducing absorption. Current estimates suggest that less than 10% of the silver ingested by humans is absorbed into the circulation [18], but this can be expected to vary widely according to the age, health and nutritional status, and composition of the diet.

Individual case studies like that conducted in a severely argyric patient following silver acetate antismoking therapy indicate that the amount of silver absorbed is low but that 18% of this is retained in the body [39]. After 2 years self administration of an antismoking remedy, this patient exhibited an estimated total body burden of 6.4 g silver with high levels of silver in forearm skin exposed to solar irradiation. Her blood silver levels increased marginally within 2 hours of administration of a radiolabelled (^110m^Ag) silver acetate lozenge, and urinary silver excretion remained fairly constant over 7 days. Studies relating to the absorption and metabolism of silver from dental amalgams show good correlations between levels of silver eluting from dental amalgams and concentrations observed in soft tissues and in blood, hair, and urine [40, 41]. The famous “blue man” of Barnum and Bailey's Circus was believed to contain as much as 90–100 g of silver in his body with deposits in bone (0.21%), muscle (0.16%), kidney (0.24%), and heart (0.15%) but the reliability of silver quantitation in tissues in 1927 is questioned [42].

Presently, toxic risks associated with silver ingestion are low since most products releasing Ag^+^ for oral or gastrointestinal hygiene have been removed from current pharmacopoeias and permitted lists in most countries in view of the risks of argyria [2, 3]. Silver is widely used in the purification of drinking water, and in the form of silver copper filters, has a major role in cleansing hospital water systems [43]. Average levels of silver in natural waters was at 0.2 *μ*g·L^−1^, and levels in drinking water (USA) that had not been treated with silver for disinfection purposes ranged from barely detectable to 5 *μ*g·L^−1^ [44]. Water treated with silver may have levels of 50 *μ*g·L^−1^, with most of this silver present in a nondissociated form as silver chloride. Estimates suggest that the silver absorbed from drinking water represents a relatively low proportion to that absorbed from the diet which ranged from 7 to 80 *μ*g daily. The World Health Organisation considered in 1996 that on the basis of present epidemiological and pharmacokinetic knowledge, a lifetime intake of about 10 g of silver can be considered the human No Observable Adverse Effect Level (NOAEL) and that the contribution of silver from drinking water would be negligible [44]. Even in special situations as when silver is added to water for maintaining bacteriological quality, concentrations of silver of 0.1 mg·L^−1^ (a concentration that gives a total dose over 70 years of 50% of the human NOAEL of 10 g) can be tolerated without risks to health [43, 44]. Greater risks are expected through uncontrolled use of colloidal silver products containing unspecified levels of ionisable silver which are commercially available in some countries as food additives, health supplements, breath fresheners, multipurpose antiseptics, topical therapies, and many other claims; many of which are not substantiated by scientific or clinical evidence [45–47].

Older studies document therapeutic administration or accidental consumption of high doses of silver nitrate as a cause of argyria and argyrosis, corrosive damage in the mouth or gastrointestinal tract, pain, and even fatality [48, 49]. Lethal oral concentrations of silver nitrate in humans have been estimated at approximately 10 g, but this is largely attributable to the strong acidity of the nitrate anion released and not Ag^+^ absorbed [2]. Blumberg and Carey reported a case of a chronically ill 33-years-old lady who had supposedly taken oral silver nitrate capsules (30 mg silver daily) on alternate periods of 2 weeks for over a year (total dose 6.4 g) and who showed greatly elevated blood silver of 0.5 mg/L (normal ca 2.3 *μ*g/L) [48]. It is unclear whether ill health was in part due to the corrosive effects of the nitrate or to other causes but she developed generalised argyria even though her argyraemia declined slightly after 3 months following withdrawal of treatment. Several other cases are reported where intentional or accidental oral consumption of silver nitrate led to gastrointestinal lesions but where the actual amount of silver ingested is not known [49]. The low systemic toxicity of oral metallic silver is illustrated by a study of 30 healthy volunteers who consumed silver leaf (50 mg/day) for 20 days [50]. Apart from transitory changes in hepatic enzymes, the treatment was well tolerated without symptoms of argyria.

Animal models provide limited predictive information concerning the gastrointestinal absorption of silver. As in humans, silver nitrate solutions are highly irritant and potentially fatal at high doses in all species due to the anion, but the uptake of Ag^+^ varies greatly. Thus rats, mice, and monkeys given silver nitrate (^110m^Ag) in drinking water absorb less than 1% of the silver administered within 1 week whereas in dogs, up to 10% may be absorbed [51]. From these experiments, the authors reasoned that a 70-kg man would retain approximately 4% of the dose administered. This extrapolation is seriously flawed on account of the imprecision of the experiments conducted and wide interspecies differences in gastrointestinal physiology and dietary requirements. Uptake and retention of silver was based upon patterns of intake and faecal excretion patterns but did not allow for secondary and tertiary excretion via the urine and hair [1, 17]. Biliary excretion is the principle route for elimination of silver from the human body.

### 2.2. Inhalation

Argyria and argyrosis provide unequivocal evidence for silver absorption following long-term inhalation of colloidal silver preparations or occupational exposures to silver or silver oxide dusts or silver nitrate [47, 52]. Detailed study of the uptake of silver through nasal and respiratory membranes has not been seen, but it is expected that inhaled silver or silver compounds ionise in mucoid secretions or alveolar pulmonary surfactants allowing Ag^+^ to be absorbed through alveolar epithelia. Silver precipitates in lungs and is absorbed by alveolar macrophages, but it is unclear to what extent Ag^+^ interacts with or is precipitated by the phospholipid content of this pulmonary surfactant, or whether this secretion acts as a barrier to absorption.

Di Vincenzo et al. evaluated 37 workers involved in silver industries and reported blood, urine, and faecal silver levels of 11 *μ*g·L^−1^, <0.005 *μ*g·g^−1^, and 15 *μ*g·g^−1^, respectively, compared to <0.5 *μ*g·L^−1^, <0.005 *μ*g·g^−1^, and <1.5 *μ*g·g^−1^ in nonsilver-exposed subjects [17]. They estimated that human exposure to the threshold limit values, set by the American Conference of Governmental Industrial Hygienists (ACGIH) and the European Union of 0.1 mg/m^3^ could lead to faecal excretion of 1 mg of silver daily, but that argyria is unlikely to occur at these exposure levels. Other research demonstrates that blood silver is much influenced by the solubility and ionisation capacity of environmental silver exposures. Thus, Armitage et al. showed that blood silver levels in melters, refiners, and silver nitrate producers exposed to soluble silver salts were in the range from 0.1 to 23 *μ*g·L^−1^ whereas those exposed to metallic silver with considerably lower ionisation potential showed argyraemias of 0.2–2.8 *μ*g·L^−1^ (control <0.1 *μ*g·L^−1^) [38]. Neither group exhibited argyria. In a similar way, 27 silver reclamation workers exposed to airborne silver halides of low solubility at 0.005 to 0.240 mg/m^3^ exhibited very low absorption with blood silver levels of 0.01 *μ*g·L^−1^ [53]. 

Occupational health reports published before the introduction of stringent health and safety at work regulations show that inhalation of silver nitrate dust is a cause of bronchitis, squamous metaplasia, and pigmentation of the respiratory tract resembling anthracosis and siderosis [54]. Rosenman et al. [33, 34] examined 27 workers in a silver plant exposed to metallic silver, silver oxide, silver chloride, and silver alloyed with other metals and reported that those exposed to environmental silver contamination levels exceeding the recommended 0.01 mg/m^3^ (i.e., 0.04–0.35 mg/m^3^) showed raised blood and urinary silver levels (blood Ag, >0.27 *μ*g·100 mL^−1^; urine Ag, >1.91 *μ*g·L^−1^), but that blood or urine silver levels did not correlate well with levels of environmental silver in work areas. A 29-years-old man accidentally inhaled dust containing ^110m^Ag and ^65^Zn in a minor nuclear reactor incident [55]. He was assumed to have inhaled about 100 nCi of each element, the specific activities being approximately 15 Ci and 3 Ci per gram respectively. Radio labelled silver was monitored in his lungs, urine, and faeces for up to 200 days. Faecal excretion persisted for at least 300 days. The half-life of silver in this patient was estimated to be 52 days.

Greater occupational risk may be experienced by those exposed to inhalation of airborne silver particles as used in impregnation of biomaterials and in plating. As Burrell demonstrated, nanocrystalline particles (<20 nm diam.) with a large surface to volume ration are expected to be dissolved more rapidly in moisture and achieve greater absorption [4]. This implies that increased silver dissolving in alveolar fluid will lead to greater lung volumes. Alveolar macrophages (dust cells) sequester a large proportion of silver particles inhaled, thereby limiting the amount available to dissolve in alveolar moisture or to invoke inflammatory changes. Reference values have not been set on this problem but some hygienists place the risk of nasal and pharyngeal inflammation at exposures as low as 0.1 mg/m^3^ with particles of grain size <20 nm diameter [56]. Much research is needed in this area. 

Extrapolation of human risk from inhalation toxicity studies of silver in animal models is complex in view of major interspecies differences in respiratory patterns and relative lung volumes [57]. Whereas absorption of silver in a dog's lung following intratracheal administration (equivalent to 1 *μ*gAg per cm^2.^ daily) is low, rats exposed to nanoparticulate silver exhibited a rapid clearance pattern [58]. Alveolar macrophages were involved in mobilisation of silver released from silver nitrate, and silver precipitates were seen in alveolar phagolysosomes. Physiopathological evaluations have indicated that in subacute (90 days) inhalation studies, rats exposed to nanosilver (18 nm diameter) for 6 hours daily the lung function was markedly depressed, and female rats exhibited a dose-related increase in inflammatory responses [59]. Thickening of alveolar membranes and granulomatous changes were reported after prolonged exposure to silver nanoparticles, but fatalities were not recorded.

### 2.3. Dermal Contact and Percutaneous Absorption

The majority of products containing silver or silver compounds for antibiotic purposes come into contact with human skin at some time, but clinical and experimental studies indicate that percutaneous absorption of silver is exceedingly low. Epidermal keratin and phospholipids of the epidermal barrier function provide effective barriers with exposed sulphydryl groups irreversibly binding free Ag^+^, in much the same way as other metallic elements [30, 60]. Where severe generalised argyria has been reported in occupational situations, it is expected that the greatest proportion of the Ag^+^ absorbed occurs through inhalation or through contamination of contaminated food and drinking water [52].

The increasing use of metallic silver, silver thread, or silver impregnates in textile fibres designed for hygiene purposes might be expected to lead to percutaneous absorption, increased blood silver, and some accumulation of silver precipitates in the skin in chronic exposures. However, risks of argyria through the use of silver antibiotics in textiles and hygiene clothing are negligible even where the skin is warm and hydrated [1]. As discussed in the recent conference “Biofunctional Textiles and the Skin” [61], concentrations of Ag^+^ released for controlling dermatophytes and superficial bacterial infections were exceedingly low and sustained. Whilst more clinical research is necessary in this area, it is noteworthy that when a wound dressing containing 85 mg·100 cm^−1^ was applied to the skin of patients with chronic ulcers for 4 weeks, blood levels of silver were not significantly different from control patients [16].

Tracer studies using ^111^Ag indicate that <4% of the total Ag^+^ released from topically applied silver nitrate solution is absorbed through intact skin [62]. Very low but perceptible penetration of nanoparticulate silver was demonstrated in human skin *in vitro* with in Franz perfusion cells [63]. Median silver concentrations of 0.46 ng/cm^2^ and 2.32 ng/cm^2^ (range 0.43–11.6) were found in the receiving solutions of cells where the solution of nanoparticles was applied on intact skin and damaged skin, respectively. Granules of inert silver precipitate were detected in the stratum corneum and the outer layers of the epidermis by electron microscopy. Silver flux in damaged skin after 24 hours was 0.62 ± 0.2 ng/cm^2^ with a lag phase of <1 hour. Penetration of silver through guinea pig skin, which is similar in thickness to human skin, was estimated to be <1% in 5 hours [64].

Clinical studies have shown that silver sulphadiazine (100 g) in an amphiphilic formulation (Flamazine) is not noticeably absorbed through intact skin but in burned patients (>5% total body surface) and that percutaneous absorption increased in line with the severity of the wounds, their depth and vascularity, and the concentration applied [35, 65]. Patients with >20% burns exhibited blood silver levels exceeding 200 *μ*g·L^−1^ with the highest concentrations at 310 *μ*g·L^−1^, without argyria. Blood silver levels increased twentyfold within 6 hours to at least 40 *μ*g/L, rising to plateau after 4–7 days. Silver nitrate is appreciably more astringent than silver sulphadiazine and ionises more rapidly when applied topically as Strong Silver Nitrate (75%), silver nitrate sticks/pencils, or douches to remove warts, callus or undesirable granulations, but Ag^+^ penetration is very low. Ag^+^ binds to epidermal keratin and blackens on exposure to solar radiation to give characteristic brown-black discoloration. Local skin discolorations rarely occur following application of sustained silver-release wound dressings and occupational contact with silver oxide and other ionisable silver compounds, but are not representative of true argyria which is long lasting. Experimental studies have demonstrated that silver precipitates in epidermal wound debris, proteins (albumins and macroglobulins) in wound exudates, or as relatively insoluble silver chloride in the skin surface exudates to be lost in normal repair processes [13].

### 2.4. Miscellaneous Routes of Silver Absorption

Silver is employed in catheters for renal drainage, central vascular insertion, intraventricular drainage in patients with hydrocephaly and cerebrospinal fluid disorders, and in in-dwelling intraperitoneal use [8]. A sustained release of Ag^+^ from metallic silver, silver sulphadiazine, or other silver compounds is necessary to achieve antimicrobial efficacy, but the amount released into the circulation for binding plasma albumins and macroglobulins is not known. Schierholz et al. [15] considered that most of this irreversibly bound silver has no toxicological, physiological, or antimicrobial significance and that silver-coating or impregnation of medical devices is only effective clinically when the concentration of free Ag^+^ is increased and the effect of contact with serum proteins and inorganic anions minimised.

Isolated cases are reported where abnormally high blood or tissue silver levels has been associated with the use of silver in patients implanted with silver-containing heart valves, bone cements, and acupuncture needles but none show silver or Ag^+^-binding to be a cause of tissue damage. A 76-year-old patient implanted with total hip replacement was shown to have blood silver 1000 times higher than normal, but this declined rapidly following the removal of the prosthesis and silver-containing bone cement [66, 67]. During the following 2 years, her serum silver concentrations decreased from >60 to 20 times higher than normal, and the patient partially recovered from an idiopathic muscle paralysis. A second interesting case of raised blood silver related to the use of silver as an antibiotic or anticalcification additive in bioprosthetic heart valves [68]. The valve was withdrawn from clinical use for reasons other than the silver inclusion or Ag^+^ release, but sheep implanted with silvered valves exhibited transitory increases in argyraemia to 40 ppb within 10 days of implant with decline to normal 30 days after surgery. Silver accumulated in the liver (16.75 mg·g^−1^ dry wt.), kidney (8 mg·g^−1^), lung, brain, and spleen (<5 mg·g^−1^) without evidence of toxicity.

Silver and gold acupuncture needles used in ancient Japanese “Hari therapy” for relief of muscular pains, fatigue, and other discomforts are potential causes of macular or more generalised argyria-like symptoms [69, 70]. Blood silver levels are not known from either report but the extent of argyria was related to the duration of acupuncture therapy and the number of needles inserted, and hence the quantity of Ag^+^ released directly into the dermis. One 57-year-old lady is recorded as implanting 2,500 needles in 13 years to alleviate symptoms of rheumatic fever.

### 2.5. Silver Metabolism

Silver absorbed into the body as Ag^+^ readily binds to intracellular proteins, notably serum albumins and macroglobulins for metabolism and distribution to bone and soft tissues. Experimental studies have demonstrated that Ag^+^ actively absorbed from silver nitrate or silver sulphadiazine induces and binds the cysteine-rich proteins—metallothioneins (MTs) I and II in metabolically active cells of the wound margin [14, 71]. MTs are major metal carrier proteins but also serve as cytoprotectants in binding toxic metal ions thereby reducing risks of cytoplasmic damage.

Controversies exist on the predominant routes of silver metabolism in the human body, its transitory or longer-term accumulation in kidney, liver, and bone, and its excretion patterns in bile, urine, hair, and nail [36, 39]. The biliary route of excretion predominates over the urinary route but urinary silver measurement may provide a convenient index of silver absorption by all routes and serve as a guide to the total silver content of the body at blood levels of <100 *μ*g·L^−1^ [36, 65]. At higher concentrations, patterns of urinary excretion are irregular. Biological monitoring of workers exposed to long-term environmental silver residues has shown raised silver concentrations in hair, blood, urine, and faeces [17, 35], but faecal silver represents that excreted in bile plus the 90% or more ingested with food and not absorbed into the circulation. From their examination of 37 silver workers, Di Vincenzo et al. concluded that at recommended environmental concentrations of 0.1 mg/m^3^ (TLV), faecal excretion of silver would be about 1 mg daily [17]. Critical evaluation of reported clinical and experimental studies has shown that silver is not absorbed into neurological tissue but is bound as inert precipitates in lysosomal vacuoles of the blood brain barrier and blood-CSF barrier [72, 73].

## 3. Manifestations of Silver Toxicity

### 3.1. Argyria

Argyria is the principle manifestation of chronic inhalation or ingestion of metallic silver or ionisable silver compounds. Excessive absorption of Ag^+^ over a long period leads to a state of silver “overload” in the circulation, where absorption exceeds the capacity of the liver or kidney to eliminate the metal in bile and urine, respectively. Argyria is characterised by the deposition of inert precipitates of silver selenide and silver sulphide in the connective tissue surrounding the vascular tissue and glands of the papillary layer of the dermis but not epidermis [52, 69, 70]. The fine deposits are inert, intracellular (lysosomally bound) or intercellular in distribution, and long lasting or permanent. The mild to profound blue-grey discolorations of the skin and nail bed occur mainly in light-exposed areas and on occasions may be severely disfiguring [19]. There is no evidence to associate argyria with cellular damage or altered sensory perception in the skin, and even in cases of profound discoloration, argyria is not life threatening. In severe cases of generalised argyria, the discolorations may be psychologically disturbing since they are not readily removed chemically or by surgical dermabrasion. Fatalities in patients with profound argyria or argyrosis have been attributed to pre-existing medical conditions and not silver-related aetiology. Argyrosis more specifically denotes silver precipitation in the cornea or conjunctiva of the eye and is regarded by some as a more sensitive indicator of silver exposure [33]. Whilst argyria is an unequivocal manifestation of chronic exposure to silver, on occasions, raised blood silver levels (argyraemias) are reported in silver workers where overt discoloration of the skin, eyes, mucus membranes, or nail bed are not recognised [35, 53].

The mechanism for dermal argyria is not fully understood but is thought to relate to imbalances in the local concentrations of soluble and insoluble complexes of silver in the middle or upper dermis and the action of lysosomal reductase [74]. Buckley and Terhaar argued that in the mid-dermis, insoluble silver appears to be in an equilibrium with a variety of soluble forms of silver such as Ag^+^ and silver mercaptides rich in cysteine and homocysteine, both of which are soluble at >pH 7. They considered that an increase in soluble silver content involves the formation of more insoluble silver by a reductive process represented by the equilibrium


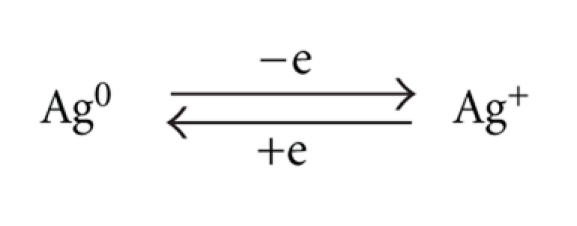


Formation of insoluble silver precipitate in dermal granules has been attributed to lysosomal action with the concentration of silver in lysosomes estimated to be 10^−5^ M. They estimated that this molar concentration is “similar to the amount required to maintain oxidative stability of silver in dispersions such as Argyrol (a colloidal mild silver protein)”. They also suggested that a critical concentration of soluble silver exists, below which any lysosomal reductase is inactive, and where silver remains solubilised. Whilst these assertions have not been challenged, more recent work implicates selenium as a central factor in the production of insoluble silver complexes in argyric states. Microanalytical studies have revealed a tenfold increase in selenium in skin biopsies of argyric patients and have implicated the element in the “detoxification” of silver [75]. X-ray emission spectroscopy of inert silver deposits in the kidney of a patient with generalised argyria showed that the particles were composed of silver selenide (Ag_2_Se) and not silver sulphide, suggesting that selenium was replacing the sulphide moiety [76, 77]. Silver selenide is a highly insoluble nontoxic material and not associated with reactive changes in tissue biopsies.

Despite electron microscopic evidence to the contrary [74, 78], silver deposited in connective tissue around hair follicles, and sebaceous or eccrine glands do not migrate across the dermoepidermal interface to be excreted in desquamated keratinocytes or in glandular secretions [52]. Small amounts of silver sulphide deposited intercellularly in the dermis can be expected to be phagocytosed by dermal macrophages or eliminated through normal tissue repair process [79, 80], but in cases of generalised argyria the discolorations of the skin, buccal membranes, and hair and nail bed are expected to be long lasting or permanent. Whereas blood silver levels are usually greatly elevated in clinical argyria, argyraemia is not a reliable guide. Thus, Coombs et al. showed that patients treated with silver sulphadiazine for severe burns and showing blood silver of >300 *μ*g·L^−1^ failed to show skin discoloration [65] whereas other cases of overt argyria failed to show correlation with elevated blood silver [81].

Melanin granules in the skin may protect against argyria by absorbing solar energy, but silver is not known to influence either melanocyte function or melanogenesis [52]. On rare occasions where argyric discolorations have been erroneously identified as melanoma-like lesions, silver sulphide (or silver selenide) deposits were attributable to Ag^+^ leaching from silver sutures used earlier in eye surgery [82, 83].

### 3.2. Argyrosis (Argyrosis Conjunctivae)

Argyrosis is defined as a dusky grey/blue pigmentation of the cornea and conjunctiva resulting from deposition of inert silver precipitates following chronic occupational, therapeutic, or environmental exposure to silver or soluble silver salts [33, 81, 84]. It is frequently a more sensitive outward sign of silver exposure than argyria, but like argyria it is not associated with pathological damage in any tissue. Therapeutic argyroses may have been known since the 17th Century when silver nitrate was used to treat epilepsy and venereal diseases and in the late 19th century when Credé et al. introduced silver nitrate as a prophylactic for neonatal eye disease [85], but more recent reports relate more to chronic occupational exposures to silver or the use of colloidal silver preparations for ocular infections [81, 84, 86, 87]. Exposure to silver in soldering, in particular, is held to be a common cause of argyrosis, but less commonly the use of silver in eyelash tints, jewellery work, therapeutic use of silver nitrate or colloidal silver preparations (notably 1% Argyrol, mild silver protein in eye drops), and industrial accidents are reported [88–93]. In each case, silver precipitates in the form of sulphide or selenide have been reported in the cornea, conjunctiva, lens, and lachrymal tissues with the severity reflecting the duration and severity of exposure, the ionisation of the silver compounds involved, and the nature of the exposure. The occupational risks of argyrosis and the toxicophysiological significance of the cytological changes are well illustrated in a study of 30 workers in silver nitrate and silver oxide manufacture [33, 81]. The conjunctiva was most frequently involved (20/30) with corneal pigmentation in 15 workers following a mean exposure period of 5 years. Slit lamp and visual acuity tests revealed more profound pigmentation of the caruncle and semilunar folds of the conjunctiva, and with corneal changes affecting the limbus, Descemet's membrane and peripheral cornea. Lens changes were noted in 4 patients. Electrophysiological tests revealed a lack of retinal damage in all volunteers, but 10 reported decreased night vision and a significant association between this nyctalopia and pigmentation of the conjunctiva/cornea. Cauterisation using lunar caustic and the use of silver nitrate to stem haemorrhages have been associated with corrosive damage and corneal opacities mainly attributable to the caustic action of the nitrate anion [92, 94]. On occasions, discolorations attributable to silver precipitates in conjunctiva, cornea, or lachrymal gland tissues have simulated melanoma, but correct diagnoses have been provided by biopsy examination and records of patients' clinical histories [82, 83, 95].

Correct diagnosis of argyrosis has been aided by confocal and specular microscopy, X-ray analysis, and electron-microscopy, but the deposition of silver precipitates in various circumstances has varied greatly according to the type of exposure and the ionisation patterns of the silver compounds implicated. In a study simulating Credé's preventive therapy with 1% silver nitrate therapy for neonatal eye infections (*ophthalmia neonatorum*), a lamellar keratectomy from a very young treated child revealed electron dense granules 100–300 nm in diameter deep in the corneal stroma [96]. In contrast, in an accident victim exposed to silver nitrate in an explosion, silver precipitates were more widely distributed in the eyelids, conjunctiva, and superficial layers of the cornea with diffuse particles located in the epithelial basement membrane, Bowman's layer, and Descemet's membrane [90]. Silver-rich particles in the deep corneal stroma were lysosomally bound in connective tissues or free within intercellular spaces and associated with tissue debris, similar to those reported in silver-related dermal injuries. The mechanism of argyrosis has not been investigated in detail, but it is expected to follow a similar pattern to that in argyria [86, 87]. High-selenium and silver-intracellular precipitates were prominent in the region of the rough endoplasmic reticulum as demonstrated using X-ray microanalysis with energy dispersive technology (EDAX) [86].

### 3.3. Silver in Soft Tissues

The skin (and its appendages), eye, brain, liver, kidney spleen, and bone marrow are listed as principle target tissues for silver deposition following systemic absorption [3, 17, 21, 33, 42]. Critical analysis of published literature revealed that despite claims of neurological damage in clinical and experimental studies, silver is not absorbed into the brain and central or peripheral nervous systems, and there is no substantive evidence that it passes across either the blood brain barrier or blood-CSF barrier in any species [72, 73]. Silver acetate when used as deterrent to smoking evokes a bitter taste in the presence of tobacco smoke but is otherwise safe and effective [39]. Elsewhere, Westhofen and Schafer [79] presented a case of a patient with generalised argyrosis associated with progressive taste and smell disorders, vertigo, and hypesthesia. The findings were ratified by chemosensory and electrophysiological tests, and hypogeusia and hyposomia were checked using subjective and olfactory tests but apart from electron-dense deposits of silver sulphide in macrophages and along perineuria and nerve tracts no histological damage was recorded using light or electron microscopy.

Clinical and experimental studies regularly list the liver as the principle organ for silver accumulation and elimination, but apart from transitory changes in certain metabolising enzymes, no evidence is seen to show that even in patients with blood silver of >200 *μ*g·L^−1^ or advanced argyria, silver is a cause of irreversible pathological hepatic damage [65, 97, 98]. Daily administration of 50 mg silver leaf to 30 healthy volunteers for 20 days led to transitory increases in blood phospholipid, triglycerides, cholesterol, glycaemia, and associated enzymes, but no functional changes in the tissue [50]. Electron microscopy has confirmed that in patients with high hepatocellular silver deposition (up to14 *μ*g.^−1^ wet weight), the precipitates are inert, lysosomally bound, and presumably extruded into bile ducts as a normal physiological process [65, 99]. Experimental studies in animal models have shown variations in hepatic management and biliary excretion of silver. Intravenous injection of dilute silver nitrate was associated with biliary excretion patterns of 0.25 *μ*g·kg^−1^/min. in rats, 0.05 *μ*g·kg^−1^/min. in rabbits, and 0.005 *μ*g/.kg^−1^/min. in dogs [100]. As in other tissues, Ag^+^ evokes and binds MT-1 and MT-2 and is eliminated innocuously in bile without morphological change [101]. Subacute (28 day) toxicity tests showed that rats tolerated massive doses of 1000 mg·kg^−1^ nanoparticulate silver (60 mn) without significant changes in body weight, but that at doses of >300 mg·kg^−1^, increased alkaline phosphatase and cholesterol levels may reflect functional liver changes in the tissue [102]. Klaassen confirmed that faecal excretion is greatly superior to biliary elimination for silver and that in ^110m^Ag-labelling experiments, bile concentrations within 2 hours of intravenous administration were 16–20 times higher than in plasma, reflecting clear dose-related plasma to bile gradients [103]. Faecal excretion accounted for 70% of the administered dose compared to <1% in urine. The study also emphasised marked interspecies differences in silver metabolism and excretion, with rabbits excreting the metal at a tenth of the rate seen in rats and dogs at one hundredth of the rate. Experimental studies in rats have also demonstrated that copper and the antioxidants selenium and vitamin E can influence the hepatobiliary transport and retention of silver in the liver [104–106]. Copper and silver are known to interact in MT and caeruloplasmin binding whereas selenium exhibits a strong tendency to precipitate silver as silver selenide, thereby promoting silver retention in the tissue [105].

Urinary excretion of silver is appreciably lower than biliary elimination and provides a less accurate measure of silver absorption by all routes. Clinical studies in burn-wound patients treated chronically with silver sulphadiazine suggest that a “threshold” of about 100 *μ*g·L^−1^ blood silver exists and that above this level urinary secretion is variable [36, 65]. In a severely argyric patient, 18% of an oral dose of ^110m^Ag was retained in the body for up to 30 weeks [39]. The argyraemia in this patient 2 hours after treatment was low (4.5 × 10^–4^% of the dose administered), and a small proportion of the original dose was excreted in urine over the next 7 days. Neither study provided evidence of renal damage or functional impairment in a total of 23 patients subjected to known concentrations of silver. In contrast, mild increases in renal N-acetyl-B,D -glucosaminidase were reported in 4 of 27 workers exposed to silver occupationally, but the significance of this change is unclear [34]. Other occupational health studies confirm renal management and excretion of silver with no obvious pathological effects [17, 33, 34, 53].

Renal pathology is an expected risk where silver nitrate is instilled into the renal pelvis or ureter as a therapy for filarial worm infestations like *Wuchereriay brancrofti* (chyluria), which represent major health risk in Southeast Asian countries [107–109]. Renal and hepatic failure, acute necrotizing ureteritis, obstructive nephropathy, and papillary necrosis have been reported following injection of up to 3% silver nitrate, although practitioners consider that the therapy is a “safe and minimally invasive treatment” for chyluria [107, 110].

The low nephrotoxicity of silver in the urinary tract has been confirmed in experimental studies in rodents given silver nitrate intravenously or in drinking water [111]. Silver precipitates have been observed on glomerular basement membranes, arteriolar endothelia and elastic laminae, without obvious structural damage [112, 113]. Berry et al. noted high levels of renal selenium sulphur and silver in the precipitates in renal membranes and interpreted the role of selenium as a cytoprotective agent [111]. As in human studies, precipitates of silver sulphide or silver selenide were lysosomally bound. Renal toxicity was not recorded in mice given high doses of 65 mg·kg^−1^ silver nitrate daily for up to 14 weeks [100].

The ability of silver sulphadiazine (SSD) and silver nitrate to evoke changes in white blood cell populations (WBC) following therapy in burn wounds is equivocal [114, 115]. Since its introduction in 1968 and clinical marketing in the USA in 1973 [116], SSD has been frequently documented as a cause of leukopenia and lowered granulocyte counts, but these changes have normalised when therapy has been discontinued. Thus, Thomson et al. [117] recorded reduced white blood cell counts (WBC) of ≤5000/mm^3^ in burn-wound patients treated with either SSD or silver nitrate within 3 days of injury (40 of 84 after SSD and 13 of 30 following silver nitrate therapy). Choban and Marshall confirmed this risk in patients with 15% total body surface burn wounds, but demonstrated that WBC normalised when SSD or silver nitrate therapy was withdrawn with no further complications [118]. In their view, SSD-induced leukopenia should be viewed as a “self-limiting phenomenon that does not increase the incidence of infectious complications nor affect the final outcome”. Nevertheless, burn-wound strategies should predict that where WBC fall to less than 2000/mm^−3^, SSD therapy be terminated as a precaution [119]. The mechanism for postburn leukopenia is imperfectly understood. Whilst *in vitro* and experimental studies in mice indicate that SSD can suppress leukocyte progenitor cells in the bone marrow [120, 121], in a clinical situation SSD may be just one contributory factor. Burn stress or silver allergy may be partly responsible [121–123]. Leukopenia/neutropenia was reported in nine patients with a mean WBC of 2,680/mm^3^ where SSD was associated with immature band forms in peripheral blood, but WBC normalised within 2-3 days [124]. Prospectively, a survey of opinion in 101 burn clinics in North America and review of published case reports indicated that postburn leukopenias attributable to SSD, silver nitrate, or other causes “hold little risk for the burn patient” [119].

Methaemaglobinaemia is a supposedly rare complication of 0.5% silver nitrate therapy in burn clinics, particularly in children [125]. The condition is attributable to alterations in the oxygen-carrying capacity of haemoglobin resulting from oxidation of ferrous iron (Fe^++^) to the ferric state (Fe^+++^) through the action of the nitrate anion [126], and not through Ag^+^ absorption. Nitrate is reduced to nitrite by nitrate reductase activity of intestinal flora as a preliminary to methaemoglobin formation. The condition may be life threatening, but normal blood oxygenation is restored with intravenous therapy with methylene blue [127].

### 3.4. Silver in Bone

Bone toxicity is not widely recognised in the safety evaluation of silver and silver-containing products, but there are strong indications from *in vitro *models that Ag^+^ interacts with and binds to the hydroxyapatite complex and can displace calcium and magnesium ions [128, 129]. Other research has demonstrated that Ag^+^ induces calcium release from the sarcoplasmic reticulum in skeletal muscle by acting on the calcium-release channels and calcium-pump mechanisms, presumably through oxidising sulphydryl groups [130]. Although this suggests that bone and possibly cartilage are vulnerable to prolonged release of Ag^+^ used as an antibiotic in bone cements, orthopaedic pins, dental devices, and so forth, this has not been established so far. No cases of osteoporosis have been reported following long-term ingestion or inhalation of silver or -implantation of silver-coated or impregnated orthopaedic devices. Osteoblasts cultured in the presence of silver wire failed to show a statistically significant reduction in cell growth after 48 hours, although alkaline phosphatase activity was markedly depressed [131]. Silver salts of varying solubility—oxide, chloride, sulphate, or phosphate—were entirely biocompatible with osteogenesis in cultured rabbit bone, and foreign body reactions were minimal [132]. With the exception of the oxide, all seemed to maintain the compressive strength of rabbit bone, following implantation in paraspinal muscles (Silver-coated orthopaedic fixing pins designed for external fixation were entirely compatible with bony and periosseous tissues [133]. In a clinical situation, inappropriate use of a silver-impregnated bone cement led to a 1000-fold increase in argyraemia and silver in acetabular cavity of 103.3 *μ*g·L^−1^, but these high systemic and local silver concentrations were not associated with osteological damage in this patient [66, 67].

### 3.5. Contact Allergy and Delayed Hypersensitivity

Metal allergies present major problems in diagnosis in dermatology clinics since most metals and metal salts are impure and contaminated by other metals like nickel, chromium, and cobalt with recognised risk [134, 135]. Allergy to silver is a known adverse effect of silver exposure in coinage, cosmetics, and in patients treated with silver nitrate, SSD, and sustained silver-release wound dressings to control wound infections, but a proportion of predisposed metal workers, jewellers, photographers, and other persons exposed to silver or silver salts occupationally may exhibit symptoms of delayed contact hypersensitivity [134]. The true extent of the problem is not known as diagnostic standard patch tests using 2% aq. silver nitrate are not routinely conducted except in health threatening situations. 

Argyria or overt skin reactions do not arise through contact with metallic silver, but small amounts of Ag^+^ released in the presence of skin exudates or moisture are sufficient to evoke symptoms of contact sensitivity in predisposed persons [134]. Aged solutions of silver nitrate with greater ionisation were shown to be appreciably more allergenic than freshly prepared reagents [136]. Although argyria as a response to silver and colloidal silver preparation had been reported from the beginning of the 20th Century [137, 138], the first reported case of true allergy was seen in a 26-year-old man following use of colloidal silver (Argyrol) to treat asthma and hay fever [139]. Silver allergy was confirmed using scratch tests and intradermal injection of 0.05 ml of 1% silver nitrate. In more recent times, contact allergies to Ag^+^ have been confirmed in occupational situations, and such conditions as “silver-workers finger”, “silver-fulminate itch” (in explosives industry), and “silver coat dermatitis” have been recognised [140–142]. Numerous cases are reported of SSD-related allergic dermatoses, with confirmation using patch and photopatch testing [143, 144].

## 4. *In Vitro* Cytogenicity, Mutagenicity, and Carcinogenicity

Cell culture systems have been developed in recent years as inexpensive means of examining intracellular metabolism of xenobiotic materials and mechanisms of cellular toxicity. *In vitro* tests have limited value in relation to *in vivo* assays discussed above for concentrations exceeding 5 ppm, and as far as I am aware, full preliminary screening for mutagenicity and carcinogenicity for silver and silver compounds has not been completed [145]. Published cytotoxicity tests and *in vivo* experience indicate unequivocally that silver is not carcinogenic in any tissue and should be placed in a “No Risk” category [146]. A large number of *in vitro *toxicity studies demonstrating the cytotoxic effects of metallic silver, silver sulphadiazine, or other silver compounds have been published in recent years, but observations in cultured fibroblasts, keratinocytes, and other human cell lines reflect the ability of Ag^+^ to react with sulphydryl groups, other protein residues, and enzymes associated with cell membranes leading to denaturation, structural damage, and mitochondrial dysfunction, in much the same way to that seen in bacterial and fungal cells [1]. Fibroblasts tend to be more sensitive to Ag^+^ than keratinocytes, but silver toxicity in cultured cells may be influenced by the age of the donor and the composition of the culture medium [147]. Human diploid fibroblasts and fresh human donor dermal fibroblasts were inhibited by short-term exposure to silver sulphadiazine and impaired proliferation was associated with marked changes in cell morphology including cytoplasmic deterioration and degeneration of nuclei and cell organelles [148]. Growth factors including platelet-derived growth factor (PDGF), epidermal growth factor (EGF), and basic fibroblast growth factor (*β*-FGF) which modulate cell proliferation, migration, and functional maturation in epidermal repair following injury have been shown to cytoprotect dermal fibroblasts from injury by silver sulphadiazine, suggesting that cells activated by growth factors are more resistant to the toxic effects of silver antibiotic [14], but this is unconfirmed by clinical or *in vivo* studies.

## 5. Reproductive Toxicity and Teratogenicity

Insufficient evidence is available presently to show that administration of silver or ionisable silver compounds in pregnancy is a cause of infertility, impaired foetal growth, or abnormal development in any species. Silver nitrate (1%) administered by intrauterine injection to 13 cynomolgus monkeys between 27 and 43 days of pregnancy caused early vaginal bleeding and termination of pregnancy but two of seven animals re-mated became pregnant again and delivered healthy offspring [149]. It is not known whether Ag^+^ passes transplacentally to accumulate in the foetus.

## 6. Nanotechnology and Nanocrystalline Silver

The antibacterial and antifungal efficacy of silver in medical devices, clothing, wound dressings, and so forth is directly proportional to the release of Ag^+^ and its availability to interact with cell membranes leading to lethality and inactivation of toxins produced [4]. Burrell has clearly identified the ionizing properties of metallic silver and silver compounds and shown that nanocrystalline particles (<20 mn diameter) exhibit a solubility of 70–100 *μ*g·mL^−1^, that is, up to 100-fold greater than metallic silver. He is of the opinion that these nanoparticles show specific physical and chemical properties which may influence their biological action. In his view, the “grain boundary region of crystals of <20 nm may represent a new state of solid matter” [4]. Differences in biological effect are illustrated in the antibacterial effects against *Pseudomonas aeruginosa *in culture and in the anti-inflammatory action of microcrystalline silver and silver nitrate in rodent models discussed above. Wound care products containing nanocrystalline silver (e.g., Acticoat, Smith & Nephew) have an acclaimed success in controlling infections in chronic wounds and ulcers [26]. However, further studies are necessary to determine whether the antibacterial and physiological effects are attributable to the silver ion *per se *or to the unique biological properties of the silver microcrystals.

Workers involved in production of nanocrystalline silver over an extended period are potentially at risk of inhaling microparticles leading to argyria and argyrosis unless stringent safety precautions (air filters, personal respirators, etc.) are followed [4]. Workers may be exposed to silver in the workplace unintentionally through hand to mouth transfer of materials or swallowing particles cleared from the respiratory tract. The National Institute of Safety and Health (NIOSH) is currently conducting research to determine the extent to which nanocrystalline silver poses a threat to exposed workers and under what circumstances and emphasising that presently no international standards have been introduced in the USA or elsewhere [150].

Specific toxicity studies and clinical trials have been conducted in relation to wound dressings including Acticoat containing nanocrystalline silver, where burn-wound patients exposed for up to 9 days exhibited increases in blood silver (56.8 *μ*g·L^−1^), which normalised to 0.8 *μ*g·L^−1^ after 6 months [151]. Clinical studies are urgently required to examine the occupational risks associated with the use of highly dispersed nanocrystalline silver in general-purpose biocides, consumer products, electronics, metallurgy, and chemical catalysis [152, 153].

Laboratory evaluations of the toxicity of nanocrystalline silver are not numerous. Some have been discussed above in relation to the cytotoxicity and genotoxicity of silver particles in cultured lung fibroblasts and glioblastoma cells [154]. Silver is absorbed into cultured cells by a pinocytic mechanism, and as in bacteria and fungi, can be expected to interact with and precipitate with cytoplasmic proteins leading to cell death. Minimal cytotoxic concentrations reflect the subcellular proteins and Ag^+^-binding sites. Cultured cells exposed to silver particles at 6.25–50 *μ*g·mL^−1^ showed altered cell shape and showed evidence of oxidative stress and increased lipid peroxidation [155]. Inhalation toxicity studies in rats conducted for Samsung Electronics Co. (Korea) have shown that the lungs and liver are principle target organs but that no effects were observable at environmental concentrations of 100 mg/m^3^ [58, 59, 156]. Exposures at 133 mg/m^3^ or 515 mg/m^3^ evoked inflammatory and granulomatous changes in the lung and bile duct hyperplasia.

## 7. Discussion

The present paper emphasises that health risks associated with systemic absorption of silver as Ag^+^ are low. Argyria and argyrosis are the principle observable changes associated with long-term exposure to ingestion or inhalation of metallic silver or ionisable silver compounds, but neither is life threatening or associated with irreversible tissue damage. It is debatable therefore whether these conditions should be classified as “toxic changes”, but it should be emphasised that severe long-lasting argyria and argyroses arising from unprotected occupational exposures to silver or unregulated consumption or inhalation of unregulated colloidal silver preparations can be profoundly disfiguring and a cause of serious psychological and personal problems and should not be dismissed as irrelevant observations [18, 19, 38, 157, 158]. I have seen no unequivocal evidence to show that silver exerts morphological damage on neurological tissues [72, 73].

Clinical experience has shown that transitory changes in hepatic and renal enzyme systems in patients exposed to high clinical or environmental silver are of minimal toxic significance [17, 33, 65]. At the moment, there is no good evidence to show that silver accumulates preferentially in bone, influences significantly calcium-modulated events in the heart, skin, and other tissues, or is a putative cause of osteoporosis, but further clinical research is required. On the other hand, there is irrefutable evidence that silver like most other xenobiotic metals, can evoke delayed contact hypersensitivity reactions and allergy in predisposed persons and this should be viewed as potential toxic hazard, albeit that the extent of the risk is not known [134]. Animal models have provided little guidance as to potential risks associated with silver exposure or sensitivity to argyria in humans, but some have provided useful information on cellular management of the metal and the role of silver-binding and carrier proteins in cytoprotection and metabolism [1, 14, 31, 71, 72]. *In vitro *toxicity studies with silver exposed to fibroblasts, keratinocytes, or other cell lines have minimal relationship to *in vivo *experiments using recognised animal models, but they are useful in confirming the lack of mutagenicity or carcinogenic change attributable to silver.

Regulatory authorities have evaluated reference standards and exposure limits to metallic silver and soluble silver compounds in occupational health situations, drinking water, medical devices, wound dressings, and consumer products based on published data on argyria and/or argyrosis as the only tangible evidence of silver exposure [2, 3, 44, 100, 146, 150]. At the moment, there are no clear guidelines from case studies or occupational health reports to indicate a clear relationship between clinical or occupational exposure to silver, blood silver levels, or minimal body silver concentrations consistent with early signs of the condition. As East et al. [39] showed in only one patient, profound argyria resulting from 2 years exposure to silver acetate antismoking device was associated with total body accumulation of 6.4 g of silver, much of which was deposited in the skin 71.7 *μ*g·g^−1^. Although dermal silver concentrations were 8000-fold higher than normal reference values [159], the patient remained in apparent good health. Elsewhere, argyria in a burn patient treated with a sustained silver-release wound dressing was associated with blood silver levels of 107 *μ*g·kg^−1^ and urinary silver of 28 *μ*g·kg^−1^, but no other changes [98]. Other studies claim that total body silver concentrations of 4-5 g can produce the clinical picture of argyria [160]. Fung and Bowen noted that in reported cases up to 1973, 365 cases of argyria had been referred to the US Food and Drug Administration and that a total body burden of 3.8 g elemental silver was required to evoke argyria [18]. It is emphasised that estimations of tissue and body silver in these older studies are considerably less accurate than those available these days using flameless thermal atomic absorption spectrophotometry or mass spectrometry with sensitivity of 1 *μ*g·L^−1^ tissue silver measurement or lower (B. Sampson, personal communication) [36]. 

Opportunities these days to study silver intake as a cause of detectable argyric change are few, since silver compounds are not legally available for oral or enteric administration (other than silver acetate in antismoking devices) in many countries, and introduction of far-reaching international health and safety standards at work regulations greatly reduces the risk of chronic occupational exposure. Current exposure limits for metallic silver and ionisable silver compounds of 0.01 mg/m^3^ in air (silver levels in drinking water 0.10 mg·L^−1^) set by the National Institute for Occupational Safety and Health and the American Conference on Governmental Industrial Hygienists in environmental exposures and drinking water regulations of <0.10 mg. Ag·L^−1^ (EPA) indicates that humans are unlikely to be exposed to sufficient silver in their food, drinking water, workplace, or in therapeutics in their lifetime to provoke symptoms of argyria [2, 3, 44, 161]. The amount of Ag^+^ released from catheters, textiles, and wound dressings using silver for permitted antibiotic purposes is very low and probably of no toxicological significance, other than being a cause of allergy [1, 61]. However, indiscriminate ingestion or inhalation of colloidal silver preparations (with unspecified concentrations of ionisable silver) for infective and non-infective conditions still presents a real risk for argyria and argyrosis and associated psychological problems [162, 163]. Older studies frequently cited in calculating reference values for silver such as those conducted by Gaul and Staud [138] and Hill and Pillsbury [137] should be viewed with caution. The former discussed incidences of argyria in a total of 70 patients exposed intravenously to varying doses of the highly toxic antisyphilitic drug silver arsphenamine (for which the chemical formula and ionisation potential are still not resolved) and undisclosed colloidal silver preparations claimed that argyria developed after a total dose equivalent to 1.84 g of silver. Since the actual formulation of colloidal silver products available these days is not known, it is even less likely that the silver content of those available in 1935 was known. Hill and Pillsbury gave a lucid account of the clinical picture of argyria [137], but lacked the facility to examine fully the implications of silver exposure and the pathological features of the condition.

Silver should not be regarded as a cumulative poison [73]. Only in cases of chronic systemic silver overload situations where excretory mechanisms become saturated, does silver deposit in an inert fashion in lysosomal or intercellular sites, unrelated to tissue damage. In these situations, selenium serves as a major protective factor in precipitating the silver in a highly insoluble and hence inert form of silver selenide. Although some of this may be taken up in lysosomes in macrophages, the deposits are essentially long lived or permanent. Available knowledge indicates that in normal healthy people, argyraemias of <3 *μ*g·L^−1^ are usual [36], and that raised levels are seen in persons occupationally exposed to the metal without suitable protective measures (face masks, etc.) [17, 33, 34, 53]. The inherent human risks of argyria through entering food chains presenting health risks to people living in areas highly polluted with silver residues from factory wastes as in the San Fransisco Bay region require urgent attention [164].
